# Physical Education

**Published:** 1893-09-09

**Authors:** 


### Physical Education.

Mr. Frederick Treves' handbook on "Physical Education" is the most sensible and practical treatise we have yet met with on a subject which, after the neglect of centuries, was suddenly dealt with in a spirit of delirious enthusiasm, and is only now beginning to be treated with consideration and care. Of the value of physical education there is no need to speak now; it is a recognised part of the curriculum of every school; the whole tendency of modern thought is to place the highest value on a good physique, and the only danger is that in our desire to bring our bodies to their highest muscular perfection we may forget that we are not all intended by nature to be athletes, prize-fighters, alpine climbers, or explorers of the Dark Continent. It is just on this point of the adapting of the exercise to the organism that the scientific exponent of physical education is bound to speak. We have, to our surprise as a nation, been forced to recognise that athletics bring disease as well as health in their train; that our long-distance runners, our record-makers and record-breakers of every kind, lay themselves open to diseases of heart and lungs which are the direct result of over-exertion. We have been forced to see that, while both work and play are necessary to health, sudden and severe alternations of work and play are absolutely dangerous; in fact, if you work hard you must play gently. It is the mental side of physical exercise that is most profitable to the brain-worker. He does not want to exercise his intellect anew on some problem of muscular science, but simply to be distracted from the thoughts that were previously absorbing him. When he can, like the jolly young waterman, "row along, thinking of nothing at all," he is in the condition most suited to mental and physical recuperation. It is probable that the sudden and apparently steady popularity of that "good old-gentlemanly vice," the game of golf, is due to its combining moderate exertion with complete mental distraction. The exercise is little more than walking, and an outsider is likely to echo the opinion of the young lady who "would have enjoyed the game just as much without the little ball"; but walking does not draw the mind from bacteria to bunkers, from politics to putting, from the difficulties of finance to the dangers of foozling.

The chief value of Mr. Treves' work is that it gives a very fair estimate of the relative values in physical education of the different kinds of sport. In his estimate he is scientific, but not merely scientific. He says: "In estimating the actual value of the work done in any physical pursuit, or in attempting to express what is meant by 'excessive' or 'unsuitable' in relation with muscular labour, I have been unable to make any use of the physiological method of measuring work by 'foot-tons.' This mode of measurement is no doubt of value to the physiologist, but to those concerned in physical education it is practically useless. Many of the results do not accord with what would be inferred from practical experience, nor can they be put to any practical use. The amount of muscular expenditure incurred in rowing one mile at racing speed is said to be represented by 18·56 foot-tons. But walking a mile at an ordinary pace causes an expenditure of 17·67 foot-tons, from which it must be inferred that there is very little difference between these two forms of exercise, so far as the use of the muscles is concerned. But everyone knows that the result of these two forms of exercise is very different." For, as a matter of fact, the results of exercise, beneficial or the reverse, are not to be measured in this way. Physical exertion throws into the circulation a certain quantity of waste products. When too much is not launched at once to be carried off by the usual organs of elimination the result is good—the organism is strengthened and purified. But if too much is put into the circulation at one time, it remains in the blood and forms a sort of spontaneous poison, by which the overstrained man whose rash exertion has caused it infects himself. Thus exercise is a matter which concerns not the muscles only, but the heart, lungs, liver, kidneys, nerves, and blood—in short, the whole man. It is for lack of knowing this that young people so often come back from a holiday in worse health than they went away. They have gone straight from study or desk, and instead of graduating their exertion so that the internal organs might be strengthened by degrees to carry off the waste muscular products evolved, they have at once walked, rowed, climbed, or bicycled as if they were professional athletes in full training. Thence results a tired and feverish condition in which the hapless fellow who has been looking for increased health is liable to catch any disease that lurks near. These are dangers which are little known or appreciated. A knowledge of them and of what is necessary to gain sufficient exercise without incurring risk is useful alike to physician and to the public generally. Therefore, while we commend Mr. Treves' treatise to the doctor, who may be asked at any moment what exercise is most suitable to a growing girl, an over-studious youth, or a middle-aged man, we advise that it should be placed, as an appendage to the Badminton Library, on the shelves of all who love and take exercise—and that is nearly the whole British nation.

"Physical Education." By Frederick Treves, F.R.C.S., Surgeon to, and Lecturer on Anatomy at, the London Hospital; Member of the Board of Examiners of the Royal College of Surgeons; Vice-President of the British College of Physical Education. (London: J. and A. Churchill, 11, New Burlington Street. Price 2s. 6d.)

